# TB notification rates across parliamentary constituencies in India: a step towards data‐driven political engagement

**DOI:** 10.1111/tmi.13574

**Published:** 2021-05-04

**Authors:** Geeta Pardeshi, Weiyu Wang, Julie Kim, Jeffrey Blossom, Rockli Kim, S. V. Subramanian

**Affiliations:** ^1^ Department of Community Medicine Vardhman Mahavir Medical College and Safdarjung Hospital New Delhi India; ^2^ Takemi Program in International Health Harvard T.H. Chan School of Public Health Boston MA USA; ^3^ Harvard Center for Population and Development Studies Cambridge MA USA; ^4^ Center for Geographic Analysis Harvard University Cambridge MA USA; ^5^ Division of Health Policy & Management College of Health Science Korea University Seoul South Korea; ^6^ Interdisciplinary Program in Precision Public Health Graduate School of Korea University Seoul South Korea; ^7^ Department of Social and Behavioral Sciences Harvard T.H. Chan School of Public Health Boston MA USA; ^8^ Honorary Senior Fellow National Institution for Transforming India (NITI) Aayog, Govt. of India India

**Keywords:** political commitment, regional variation, TB elimination, Geographic Information System (GIS) mapping

## Abstract

**Objective:**

National averages obscure geographic variation in program performance. We determined Parliamentary Constituency (PC)‐wise estimates of TB notification to guide political engagement.

**Methods:**

We extracted district‐level TB notification data from the 2018 annual TB report. We derived PC‐level estimates by building a ‘cross‐walk’ between districts and PCs using boundary shapefiles. We described the spatial distribution of the PC‐wise estimates of Total Notification Rate and percentage of Private Sector Notification.

**Results:**

The median PC‐wise Total Notification Rate was 126.24/100 000 (IQR: 94.86/100 000, 162.22/100 000). The median PC‐wise Percentage Private Sector Notification was 18.03% (IQR: 9.56%, 26.84%). Only 16 (2.94%) PCs met the target of 50% private sector notification. Most of high notification rates in PCs were driven by high notification in public sector. There was geographic – both interstate and within state inter‐PC – variation in the estimates of these indicators. The study identified some geographic patterns of notification – high positive outlier PCs with adjoining PCs in lower deciles of notification rates, intra‐state differences in PC performance, and similarities in notification rates of adjoining PCs in different states.

**Conclusion:**

In addition to regional inequality, the study identified geospatial patterns that can aid in the formulation of suitable interventions. These include decongestion of overburdened facilities by strengthening poorly performing units. The PCs with a high percentage Private Sector Notification can act as role models for neighbouring PCs to improve private sector engagement. MPs can play a crucial role in mobilising additional resources, creating awareness, and establishing inter‐PC and inter‐state collaboration to improve TB program performance.

## Introduction

Political commitment to eliminate Tuberculosis (TB) is one of the fundamental pillars of the End TB Strategy of WHO [1]. Different from other pillars, this requires political agreement as it involves resource allocation decisions. Many developing countries with a high TB burden face a multitude of other development tasks, complicating continued prioritisation of TB elimination. As a tool to enhance political accountability of TB elimination, we introduce a TB monitoring framework using primary political geography as a unit of measurement with a focus on private sector notification. Such a framework has the potential to guide effective policy actions.

First, measuring TB burden using political units could help overcome the inadequacy of resource investment to TB programs. Notwithstanding verbal and symbolic commitment by political leaders to end TB, the current level of investment falls short of the estimated budget. In case of India, the country that bears over a quarter of TB burden in the world, it is reported that a gap of 16% existed between the approved and requested budget during 2017–20 period [2]. Given the highly political nature of resource allocation decisions, aligning the interest of political leaders by using political geography as a unit of measurement could enhance their political engagement.

Second, TB metrics of political units can guide policies to address regional inequality of TB. In most countries, TB burden is concentrated in poorer parts of the country, yet these regions also face competing development tasks. In India, the burden of TB is 8.7 times higher in Uttar Pradesh compared to Kerala [3]. At the same time, net state domestic product per capita is nearly threefold higher in Kerala than in Uttar Pradesh [4]. Such inequality at the regional level implies the importance of choosing a unit of monitoring that is granular enough to capture the regional variation and simultaneously engage political leaders.

Lastly, focusing on private sector engagement could facilitate TB elimination in India where a majority of TB patients are seen in private sectors [5, 6]. The Government of India also recognises the significance of engaging the private sector which is codified in National Strategic Plan [7]. Monitoring private sector notification rates across the political units could convene political leaders’ interest to the proportion of TB burden handled in private sectors and guide tailored policy actions.

We use Parliamentary Constituencies (PCs) of India as an example of a primary political unit for TB metrics. PCs are political units from which Members of Parliament (MPs) are elected to represent at Lok Sabha, the Lower House equivalent of India. The MPs hold the direct authority to lead policy actions at national and local levels. At the national level, MPs have an important role in mobilising national financial resources, as Financial Bills are often initiated and approved by the Lok Sabha. At the local level, MPs are authorised to support program implementation and mobilise additional resources for the National TB Elimination Program (NTEP). Therefore, aligning MPs interests to TB needs could be perhaps the most efficient way to fill the resource gap.

Despite its potential to enhance political commitment, political geography has been underutilised as a monitoring and evaluation unit. Most nationally representative surveys, the ingredients for health and development metrics, only contain administrative unit identifiers such as states and districts. As political unit boundaries do not align with administrative unit boundaries, producing the PC‐wise estimates has been challenging. We aim to address the lack of TB information across political geography and share this conceptualisation to be widely adopted in other developing countries that experience low political priority to TB.

## Methods

While TB information is routinely collected at the district level in India, there have been no publicly available data at the Parliament Constituencies level. As the delimitation of PCs is carried out independently, a PC can span across multiple districts, and PC‐level estimates are not a direct aggregation of district‐level indicators. A cross‐walk derives PC‐level estimates from district‐level data by applying GIS‐based techniques [8, 9]. The method has been used to measure many relevant public health and development indicators [10, 11].

A cross‐walk assumes that TB distribution is unchanged within the district and is proportional to its population. If a PC comprises 40% of the population in district A and 60% of district B, the total notified patients in the PC are the summation of 40% patients in district A and 60% patients in district B. We generated a data set with each segment linked to unique PC and district, and calculated segment‐level population and notification, then aggregated by PC ID to derive PC‐level indicators.

### Data sources

#### TB Notification dataset for 2017

We extracted 2017 district‐wise TB data sets from the 2018 annual TB report [12]. All 689 districts participating in the Revised National Tuberculosis Control Program (RNTCP) had their TB notification data available in this report. There are 13 districts that did not have RNTCP reporting units, and cases were reported through adjacent districts’ RNTCP units. For example, Bandipore and Baramulla share the same reporting unit under Bandipore.

#### District and PC Shapefile for TB program

Both PCs and districts shapefiles are downloaded from the Community Created Maps of India (CCMA) [13]. The PC shapefile contained 543 PC polygons and was updated in 2019. The district shapefile contains 640 district polygons from Census 2011.

While there is no official up‐to‐date district shapefile, we used the Census 2011 district shapefile as a base map and made edits on the borders to match with actual districts in 2017 in the RNTCP program. New district documentation from Reserved Bank of India, and Bharat Map, a multi‐layer GIS platform with latest administrative boundaries, served as two critical references [14, 15]. *Georeferenced* and *edit* functions in Arc GIS were used to draw new district boundaries at a scale smaller than 1:1 000 000. For districts without RNTCP units, we dissolved adjacent districts’ boundaries to create an accurate representation of the RNTCP unit border. In the final data set, 676 polygons were identified.

#### Population estimates

WorldPop2015 provides a granular level of population distribution estimates in 2015 [16]. It estimated the number of people per pixel in a raster data format at an approximately 100 m spatial resolution in India using demographics data and land cover remote sensing imagery analysis [9]. The TB report also provided population estimates in 2017 at the district level.

### Procedures

First, we used ArcGIS Pro *intersect* to create a new shapefile based on the PC and district shapefile, identifying 3828 segments linked with PC and district ID. Next, we performed *Zonal Statistics* using WorldPop as the underlying data raster to derive the segment and district‐level population estimates in 2015. Finally, we calculated the percentage of the population by dividing the segment population by the district population.

**Figure 1:**
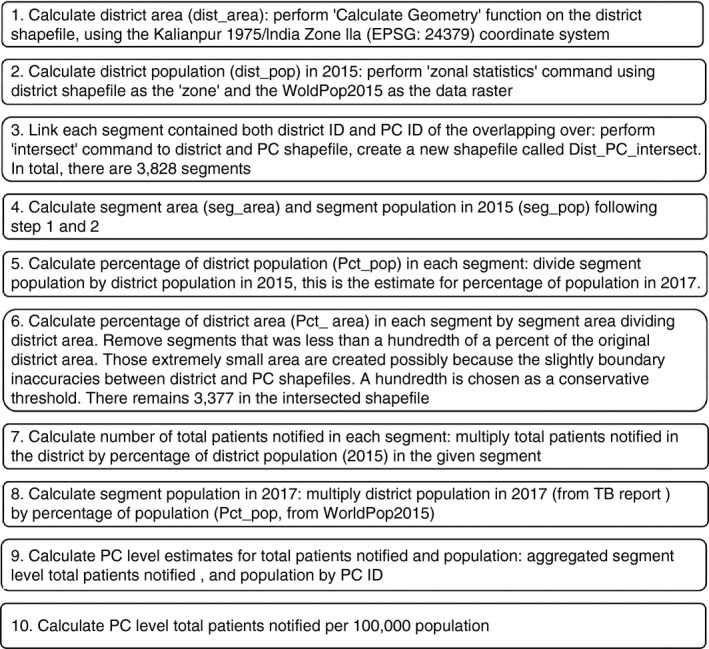
Process of cross‐walk to calculate PC level TB notification rate.

Assuming population distribution unchanged between 2015 and 2017, segment population in 2017 was calculated by percentage of the population multiplying district population in 2017. TB notification at segment level was calculated by percentage of the population multiplying district notification. The final PC‐level population and notification were aggregated by segment population and segment TB notification. A flow chart detailing the steps to derive the total notification rate per 100,000 population at PC level is shown in Figure 1.

### Ethics

The Harvard T.H. Chan School of Public Health Institutional Review Board reviewed this study and considered it exempt from full review because it utilises an anonymous public use data set.

### Indicators

We estimated TB notification rate (per 100 000 population) and percentage of private sector notification for each PC. These indicators have been recommended for monitoring the program implementation at the subnational level [17]. We used density plots to summarise the estimates and generated maps to visualise PC‐wise indicators’ geographic distribution. We used scatter plots and correlation coefficients to assess the relationship between public and private sector notification.

## Results

**Figure 2:**
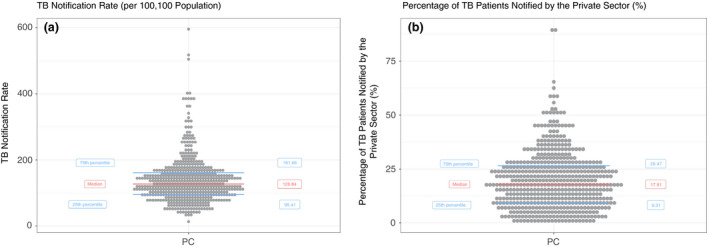
Distribution of (a) PC‐wise Total Notification Rates (per 100 000) (b) Percentage of private sector notification (%).

**Figure 3:**
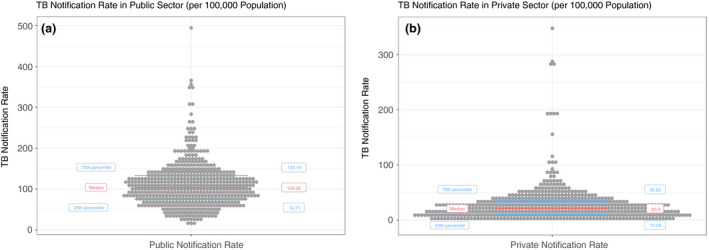
Distribution of (a) PC‐wise Notification Rates in Public Sector (per 100 000) (b) PC‐wise Notification Rates in Private Sector (per 100 000)

From the analysis, we were able to identify TB notification indicators for all 543 PCs of India. Figure 2 shows the summary statistics of the PC‐wise estimates. The median of the PC‐wise Total Notification Rates was 126.84/100 000 (Inter‐quartile range: 95.41/100 000, 161.66/100 000). The median PC‐wise percentage Private Sector Notification was 17.91% (IQR: 9·31%, 26·47%). The median notification rate in the public sector [104.25/100 000 (IQR: 74.71/100 000, 133.16/100 000)] was nearly five times more than the median notification rate in the private sector [20.9/100 000 (IQR:10.84/100 000, 35.82/100 000)]. (Figure 3).

### Total notification rate

**Figure 4:**
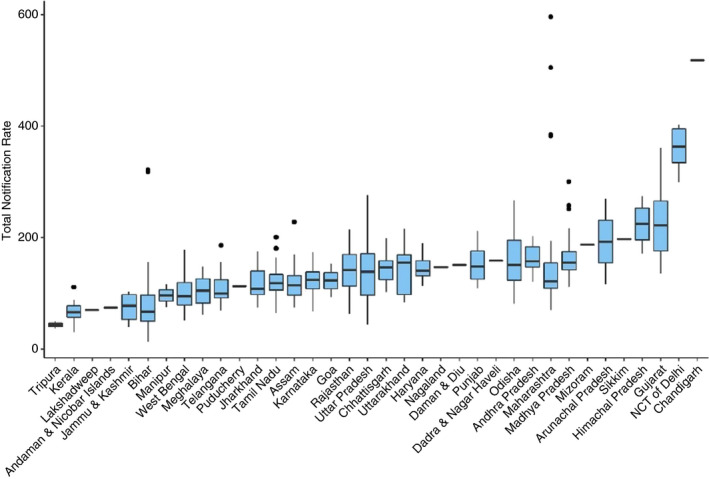
Boxplot of PC‐wise Total TB Notification rate per 100 000.

We found a large variation in PC‐wise total notification rates between states and PCs. (Figure 4). The states/UTs with the high median for PC‐wise Total Notification Rate were Chandigarh (518.09/100 000), Delhi (363.29/100 000) and Gujarat (221.80/100 000). States with lower median Total Notification Rates were Tripura (43.81/100 000), Kerala (65.76/100 000) and Bihar (67.03/100 000). Positive outlier PCs were noted in Assam, Bihar, Maharashtra, Madhya Pradesh, Tamil Nadu and Telangana. States with the greatest number of PCs in the top decile were Gujarat, Uttar Pradesh, Delhi and Maharashtra. States with the highest number of PCs in the bottom decile of the PC‐wise Total Notification Rate were Bihar, Kerala and Uttar Pradesh. (Table 1).

**Table 1:** PCs in the bottom and top deciles for Total TB Notification Rate in India

States Name (No. of PCs in decile category/Total PCs in the state)	Bottom decile (*n* = 55)		Top decile (*n* = 54)	
	*N*	Names	*N*	Names
Assam (PC: 14)	0		1	Dibrugarh
Bihar (PC: 40)	20	Arrah, Banka, Begusarai, Buxar, Hajipur, Jhanjharpur, Karakat, Katihar, Kishanganj, Madhepura, Madhubani, Muzaffarpur, Nalanda, Paschim Champaran, Purvi champaran, Sasaram, Sheohar, Supaul, Vaishali, Valmiki nagar	2	Pataliputra, Patna sahib
Chandigarh (PC: 1)	0		1	Chandigarh
Delhi (PC: 7)	0		7	Chandni Chowk, East Delhi, New Delhi, North‐East Delhi, North‐West Delhi, South Delhi, West Delhi
Gujarat (PC: 26)	0		15	Ahmedabad East, Ahmedabad West, Banaskantha, Bharuch, Chhota Udaipur, Dahod, Gandhinagar, Kheda, Mahesana, Panchmahal, Patan, Sabarkantha, Surat, Surendranagar, Vadodara
Himachal Pradesh (PC: 4)	0		2	Kangra, Shimla
Jammu & Kashmir (PC: 6)	3	Anantnag, Baramulla, Srinagar	0	
Karnataka (PC: 28)	1	Hassan	0	
Kerala (PC: 20)	11	Alappuzha, Idukki, Kannur, Kottayam, Kozhikode, Malappuram, Palakkad, Pathanamthitta, Ponnani, Vadakara, Wayanad	0	
Madhya Pradesh (PC: 29)	0		4	Bhopal, Dhar (st), Gwalior, Indore
Maharashtra (PC: 48)	0		6	Mumbai North, Mumbai North‐Central, Mumbai North‐East, Mumbai North‐West, Mumbai South, Mumbai South ‐Central
Meghalaya (PC: 2)	1	Tura (st)	0	
Odisha (PC: 21)	0		4	Berhampur, Koraput, Mayurbhanj, Sundargarh
Punjab (PC: 13)	0		1	Patiala
Rajasthan (PC: 25)	2	Barmer, Nagaur	1	Bhilwara
Tamil Nadu (PC: 39)	2	Chidambaram, Cuddalore	0	
Telangana (PC: 17)	2	Mahbubnagar, Medak	0	
Tripura (PC: 2)	2	Tripura East, Tripura West	0	
Uttar Pradesh (PC: 80)	6	Azamgarh, Ballia, Domariyaganj, Ghazipur, Jaunpur, Lalganj	8	Akbarpur, Gautam Buddha Nagar, Ghaziabad, Kanpur, Lucknow, Mathura, Meerut, Mohanlalganj
Uttarakhand (PC: 5)	0		1	Hardwar
West Bengal (PC: 42)	5	Diamond Harbour, Jadavpur, Kanthi, Mathurapur, Tamluk	0	

**Figure 5:**
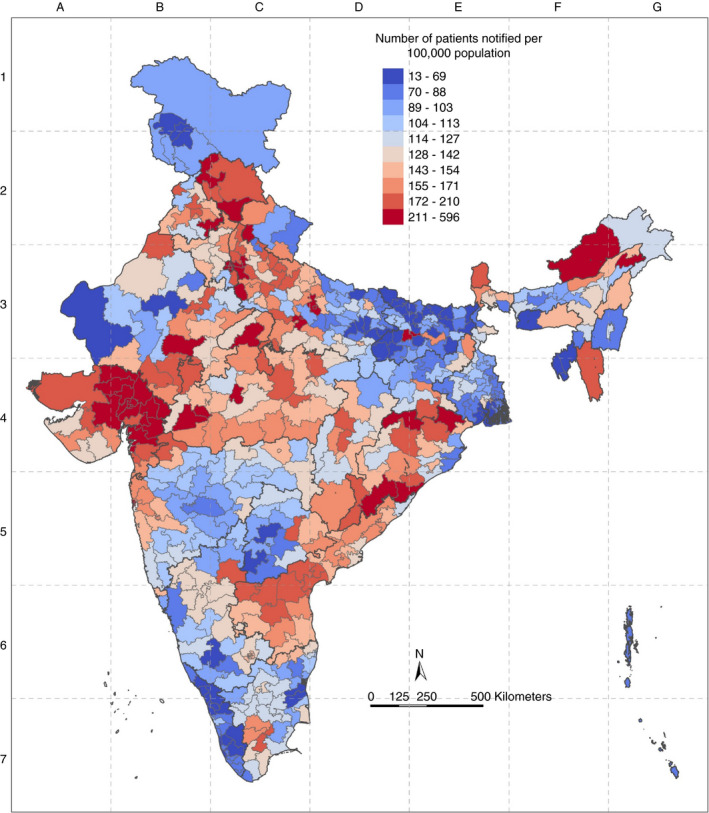
Geographic distribution of deciles of PC‐wise Total TB Notification rate per 100 000.

Considering the geographical distribution of PC‐wise estimates, we identified some pertinent patterns in Total Notification Rates (Figure 5). There was uniformity in PC performance in some states, for example in Delhi, all PCs were in the top decile, and in Tripura had both PCs in bottom decile of Total Notification Rate. In Uttar Pradesh, there was wide variation in PC‐wise estimates within the state; however, the performance of PCs was similar to PCs from adjoining states. While the positive outlier in Bihar was surrounded by PCs in lower deciles, the positive outliers in Gujarat (Mehsana) and Maharashtra (Mumbai) did not show this pattern. In Bihar, all PCs were in the lower half of the distribution, with only the state capital (Patna) and its adjoining city (Pataliputra) in the top decile.

### Percentage of private sector notification

**Figure 6:**
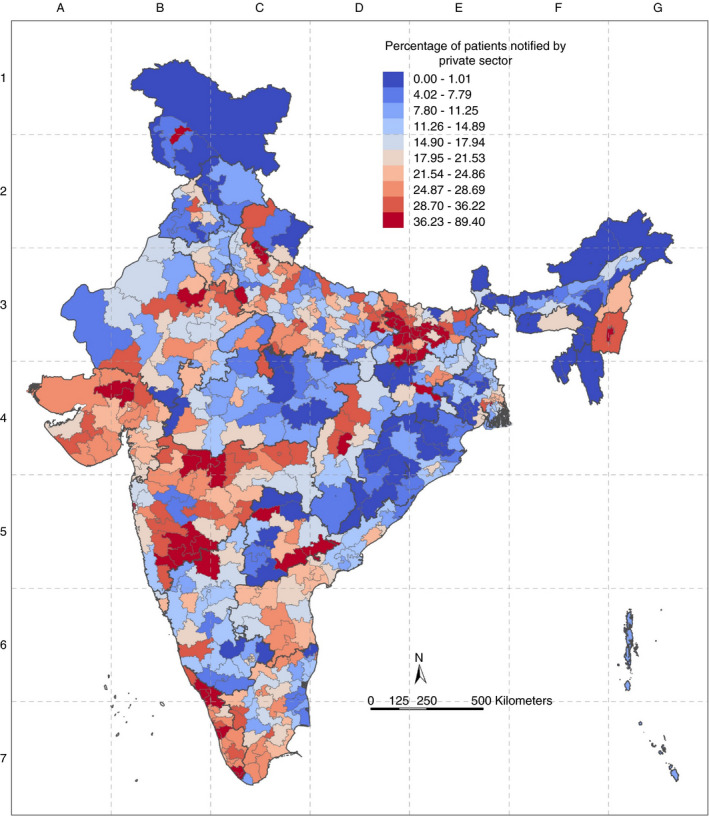
Geographic distribution of deciles of PC‐wise percentage of patients notified by private sector (%) in India.

**Figure 7:**
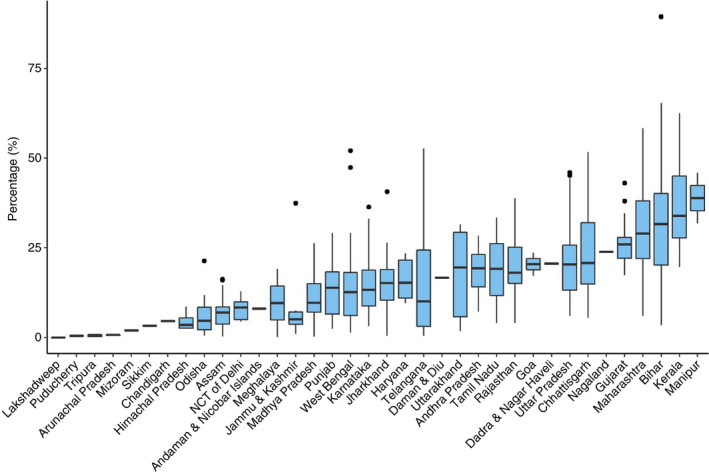
Boxplot of PC‐wise Percentage Private Sector TB Notification (%).

Only 16 PCs (2.94%) met the target of 50% private sector notification and there was a wide variation of PC‐wise percentage of private‐sector notification (Figure 6). The states with higher median values of PC‐wise percentage Private Sector Notification were Manipur (38.84%), Kerala (33.92%) and Bihar (31.61). Positive outlier PCs were noted in nine states. (Figure 7). Bihar, Maharashtra and Kerala showed the highest percentage of private‐sector notification. PCs in the bottom decile were in Odisha, West Bengal, Telangana and Madhya Pradesh (Table 2). Except for Manipur, all northeastern states had some PCs in the bottom decile of private sector notification.

**Table 2:** PCs in the bottom and top deciles for Percentage Private Sector TB Notification in India

State (No. of PCs in decile category/Total PCs in the state)	Bottom decile (*n* = 55)		Top decile (*n* = 54)	
	*N*	Names	*N*	Names
Bihar (PC: 40)	1	Jhanjharpur	15	Arrah, Aurangabad, Gaya, Gopalganj, Hajipur, Khagaria, Maharajganj, Munger, Nawada, Pataliputra, Patna sahib, Samastipur, Saran, Siwan, Ujiarpur
Chhattisgarh (PC: 11)	0		1	Raipur
Gujarat (PC: 26)	0		2	Mahesana, Patan
Himachal Pradesh (PC: 4)	2	Hamirpur, Kangra	0	
Jammu & Kashmir (PC: 6)	2	Ladakh, Udhampur	1	Srinagar
Jharkhand (PC: 14)	3	Chatra, Palamu, Singhbhum	1	Ranchi
Karnataka (PC: 28)	2	Kolar, Tumkur	1	Bijapur
Kerala (PC: 20)	0		8	Attingal, Chalakudy, Ernakulam, Kannur, Kozhikode, Thiruvananthapuram, Vadakara, Wayanad
Lakshadweep (PC: 1)	1	Lakshadweep	0	
Madhya Pradesh (PC: 29)	5	Damoh, Mandla, Ratlam, Rewa, Tikamgarh	0	
Maharashtra (PC: 48)	0		13	Buldhana, Hatkanangle, Jalgaon, Madha, Mumbai North, Mumbai North‐Central, Mumbai North‐East, Mumbai North‐West, Mumbai South, Mumbai South ‐Central, Raver, Sangli, Solapur
Manipur (PC: 2)	0		1	Inner Manipur
Meghalaya (PC: 2)	1	Tura (st)	0	
Mizoram (PC: 1)	1	Mizoram	0	
Odisha (PC: 21)	9	Bargarh, Bolangir, Dhenkanal, Jajpur, Kandhamal, Keonjhar, Koraput, Nabarangpur, Sundargarh	0	
Puducherry (PC: 1)	1	Pondicherry	0	
Punjab (PC: 13)	1	Sangrur	0	
Rajasthan (PC: 25)	1	Banswara	1	Sikar
Sikkim (PC: 1)	1	Sikkim	0	
Tamil Nadu (PC: 39)	1	Tiruvallur	0	
Telangana (PC: 17)	5	Adilabad(st), Malkajgiri, Medak, Nagarkurnool, Peddapalle	3	Khammam, Nalgonda, Nizamabad
Tripura (PC: 2)	2	Tripura East, Tripura West	0	
Uttar Pradesh (PC: 80)	0		5	Ghosi, Kanpur, Mathura, Moradabad, Nagina (sc)
Uttarakhand (PC: 5)	1	Almora	0	
West Bengal (PC: 42)	8	Alipurduars, Asansol, Bardhaman‐durgapur, Bardhaman purba, Ghatal, Jalpaiguri, Jhargram, Medinipur	2	Kolkata Dakshin, Kolkata Uttar

### Correlation between public and private sector notification rates

**Figure 8:**
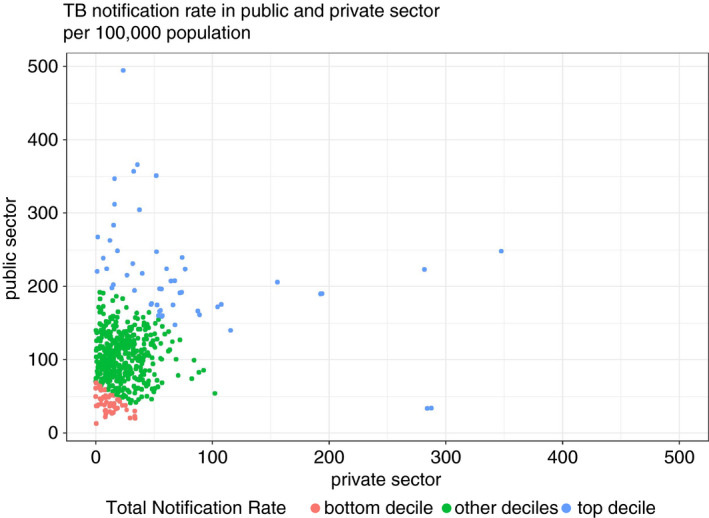
Correlation between PC‐wise notification rates in the public and private sector.

There was a significant but weak positive correlation between the public and private sector TB notification rates. The Pearson's correlation coefficient was 0.20, with a *P*‐value less than 0.001; the Spearman's correlation coefficient was 0.19, with a *P*‐value less than 0.001. Most high notification rates in PCs were driven by high notification in public sector, but there were a few PCs (in lower right corner) with low public notification and high private notification (Figure 8). PCs in bottom decile of total notification rates had lowest notification rates in both public and private sector.

## Discussion

This is the first study to estimate the burden of TB at the PC level. PC‐level data of TB program indicators will be crucial in sensitising the MPs to TB scenarios and program performance in their PCs. The Central TB Division publishes annual reports with aggregate data at the district level. However, the administrative districts and parliamentary constituencies are not coterminous. We have overcome this problem by deriving PC‐wise estimates for notification rates using the available district‐level program data by applying a cross‐walk between districts and PCs. The distribution and geospatial comparison of the estimates of PC‐wise TB notification rates have identified patterns that can lead to effective policy action.

The public health sector is the primary component of TB notification. For PCs in the bottom decile of notification rates, the strategy should be to review and strengthen the performance of the public sector, improve community awareness, followed by enhanced private sector engagement. India accounts for 26% of the total estimated global gap between incidence and notifications [18]. This strategy will help address the issues of underdiagnoses and underreporting, which are responsible for this gap.

The distribution and comparison of PC‐wise total notification rates highlight the inter‐state and inter‐PC inequalities and bring up different patterns. We noted high total notification rates and high percentage of private‐sector notification in Gujarat and a pattern of high total notification rate with a low percentage private sector notification in Delhi, Chandigarh and Himachal Pradesh. The latter indicates an overburdened public sector and the need to prioritise private sector involvement in the program. PCs with high notification rates adjoining PCs in low notification categories could indicate cross‐district health service utilisation, thereby burdening a district’s diagnosis and reporting system. For example, all the PCs in Bihar had low notification rates except for the state capital. There are temporary movements of patients from rural areas and towns into the city for diagnostic evaluation. The high TB notification in Delhi ad Chandigarh has been attributed to the state providing diagnostic care for populations beyond their boundaries [2]. In a study, one‐sixth of patients diagnosed in Chennai reported home addresses located outside the city, mostly in nearby districts [19]. A study reported that patients from neighbouring districts and states approach the capital cities’ facilities, hoping to get better quality of services [20]. In 2019, there were 11% inter‐district and 4% interstate transfers among notified patients [2]. The program should have mechanisms to detect cross‐district treatment‐seeking along with corrective actions. Another pattern was noted in the state of Uttar Pradesh, where notification rates showed within‐state heterogeneity but the similarity with PCs in adjoining states. A scheme of inter‐state partnership will help draw a common framework for improving case notification in these PCs. Elected representatives can play a role in facilitating the need‐based allocation of additional resources and establishing coordination between PCs and states. Such data‐driven interventions will be more aligned to the local context.

While PCs in Tripura and Lakshadweep have low estimates of both indicators, PCs in Kerala and Bihar are in lower deciles of TNR and higher deciles of percentage private sector notification. However, the performances of these two states in TB programs have been documented to be different. Kerala has achieved good integration of TB services with primary healthcare services, good private sector engagement and local self‐government stewardship. It has resulted in Kerala's multi‐layered and sustained TB control model [21]. The shortage of ground‐level staff has been identified as a critical limitation leading to low notification and treatment success rates in Bihar [22].

In addition to the critical role of public health systems in TB control, the contribution of the private sector in service provision has been acknowledged since the 1990s. Improving private sector notification offers an entry point for public‐private partnerships, ensures the rational use of drugs and linkages with public health action for patients in the private sector, prevents out‐of‐pocket expenditure and improves treatment outcomes. The proportion of private sector notification indicates private sector engagement in the program. The target set for the proportion of TB patients notified by the private sector is 50% for the year 2025 [7]. In this analysis, the percentage of Private Sector Notification in most PCs was <50%. Various schemes and approaches for public‐private partnership implemented in high TB burden countries for over two decades have shown mixed results [23]. Political commitment, action and investment which have been recommended as key action points for scaling up Public‐Private Mix, have not been commensurate to the scale of the problem [24]. In India, PC‐level information on private sector performance can help secure the support of elected representatives for scaling up successful experiences and ensuring the sustainability of private sector involvement. There is evidence to suggest that instead of a centrally administered uniform model, services may be decentralised to develop locally appropriate models of partnerships with private providers [25]. PCs with adequate private sector notification can serve as role models for neighbouring constituencies. Kerala has had good private sector engagement in the program for many years. An important lesson learned from the state is its success in garnering support and collaboration with private hospital consortiums and professional medical associations. They have acted as crucial links between the public and private sectors [21]. An essential strategy for improving private sector engagement would be inter‐district/interstate collaboration for experience sharing with successful models and pilot projects in the region [26, 27]. Elected representatives can create an environment conducive to such collaborations, motivate all healthcare providers to render quality‐assured TB care in partnership with the national program and increase population‐level demand for accredited TB care services. It will go a long way to achieve universal coverage of services provided under the TB program.

A careful interpretation of the study results is needed as the estimates of notification are influenced by multiple factors. High case notification rates may suggest a higher TB burden or better access to diagnosis and care and vice‐versa [17]. It indicates that the geospatial distribution of program performance indicators should be coupled with operational research for formulating appropriate interventions. Another limitation is that we have not considered different types of TB (MDR‐TB, XDR‐TB), which is important for effective policy action. The cross‐walk methodology assumes that observations are uniformly distributed across the districts, while, in reality, it is normal that some areas have higher TB notifications than others. The raster data of WorldPop2020 provided us with a granular level of population distribution, and the weighted population in the area can partly balance out the limitation. Institutional barriers and capacity bottlenecks, along with conceptual and value gaps between policy and technical communities, have been significant challenges for evidence‐based policymaking [28]. In low‐ and middle‐income countries, the issue is further compounded by limited resources, lack of faith in data due to poor quality control and a long culture of non‐use of data for decision‐making [29]. Addressing these contextual factors and barriers will augment data‐policy interaction and lead to a more systematic political engagement in public health programs.

Experience with smallpox and polio eradication has shown that achieving such ambitious goals is more than a technical mission. They are social and political phenomena requiring careful negotiations with local public health workers, political and community leaders, and members of the general population [30, 31]. In India, the MPs hold the authority to initiate and support interventions that are customised to the needs of their respective PCs. This could include increasing financial allocation, creating awareness in the community, calling attention to cross‐district treatment‐seeking, and facilitating inter‐district/interstate collaboration. The methods used in this study can be used to calculate the PC‐wise performance of other program indicators on treatment outcome, type of TB, comorbidities, and laboratory investigations [17]. It will create an opportunity to incorporate political oversight and ensure bidirectional accountability where MPs can track progress in their respective PCs. They can be accountable to their constituents and the parliament. Like the Aspirational Districts program, the PCs’ performance can be compared, and a sense of competition between PCs can be instilled to provide momentum to the TB program. As health is a state subject under the constitution, the methods used in this study can generate estimates at the assembly constituency level for effective engagement of Members of Legislative Assembly (MLAs) in the program. In the long‐term political engagement at the local level should facilitate community involvement and multisectoral coordination to address factors outside the purview of the health sector. The novel cross‐walk method proposed in this study can be used to obtain estimates of health program indicators for political units from existing aggregate‐level data in other countries with similar challenges. Information on program indicators according to stakeholder‐specific requirements can help leverage program data for enhancing their involvement in the implementation of interventions.

## Conclusion

The variation in TB notification along with regional diversity in program performance indicates that straight‐jacket solutions may not be an effective strategy to achieve the goal of TB elimination in India. Policy and program initiatives need be tailored to meet location‐specific needs. The MPs have a sustained interface with the community with the authority to voice the concerns of their constituents. Their active involvement will give the much‐needed acceleration to the program. The study results will help align the interest of policymakers in mobilising finances, correcting regional inequality and ensuring private sector engagement. It can be a step towards the data‐driven engagement of elected representatives in NTEP.
